# A Fuzzy Logic Prompting Mechanism Based on Pattern Recognition and Accumulated Activity Effective Index Using a Smartphone Embedded Sensor

**DOI:** 10.3390/s16081322

**Published:** 2016-08-19

**Authors:** Chung-Tse Liu, Chia-Tai Chan

**Affiliations:** Department of Biomedical Engineering, National Yang-Ming University, Taipei 11221, Taiwan; g39904001@ym.edu.com.tw

**Keywords:** mobile health, smartphones, prompt, fuzzy logic, activity recognition

## Abstract

Sufficient physical activity can reduce many adverse conditions and contribute to a healthy life. Nevertheless, inactivity is prevalent on an international scale. Improving physical activity is an essential concern for public health. Reminders that help people change their health behaviors are widely applied in health care services. However, timed-based reminders deliver periodic prompts suffer from flexibility and dependency issues which may decrease prompt effectiveness. We propose a fuzzy logic prompting mechanism, Accumulated Activity Effective Index Reminder (AAEIReminder), based on pattern recognition and activity effective analysis to manage physical activity. AAEIReminder recognizes activity levels using a smartphone-embedded sensor for pattern recognition and analyzing the amount of physical activity in activity effective analysis. AAEIReminder can infer activity situations such as the amount of physical activity and days spent exercising through fuzzy logic, and decides whether a prompt should be delivered to a user. This prompting system was implemented in smartphones and was used in a short-term real-world trial by seventeenth participants for validation. The results demonstrated that the AAEIReminder is feasible. The fuzzy logic prompting mechanism can deliver prompts automatically based on pattern recognition and activity effective analysis. AAEIReminder provides flexibility which may increase the prompts’ efficiency.

## 1. Introduction

Physical activity substantially benefits physical and mental health. Sufficient physical activity can reduce the incidence and severity of many adverse diseases such as type 2 diabetes mellitus, coronary heart disease, and depression [1]. Regular and moderately intense physical activity, such as fast walking and running, can increase life expectancy and contribute to a healthy life. However, around the world it is estimated that 31% of adults are physically inactive. Improving physical activity has become an essential concern for public health worldwide [1,2].

Reminders that encourage physical activity are one method for reminding and motivating people to increase their physical activity [3]. Health care providers can deliver a prompt such as a text message, voice, or image to assist people in managing their health. Periodic prompts can be delivered at various intervals, such as daily or weekly, and they can be sent through various channels, such as a short message service or telephone. The use of prompt applications has been widely applied in health care services and has positively affected patient self-management, weight loss, and physical activity [3,4]. These studies have shown the potential of prompt applications to increase healthy behavior in health care services.

Although prompt applications have a positive response, study results also indicated that people may adapt to prompts and the effectiveness of the prompts diminishes gradually. Prompts usually gave a feedback to people at a periodic interval (e.g., daily or weekly) and some people felt tired and stopped receiving periodic prompts [3,4]. Prompts that can be sent by a counselor are an alternative way to remind people, but this needs a great deal of manpower and is time-consuming [3,4,5]. Automated prompts are a cost-effective way that can save resources. Therefore, development of automated prompts based on the situation of people may be a solution to avoid diminishing effectiveness. 

There are another two reasons, which are flexibility and independency, to develop prompts that are based on the situation of people [5]. People’s life schedules are variable. For example, a person may decide to rest the next day after a long day of exercising. A prompting system that is aware of this situation may not sent a prompt in next day. However, a periodic reminder would still remind this person to be physically active, despite the person’s intention to rest, which may cause irritation. The prompting system should be more flexible, based on the exercise performance situation. In the case of increasing physical activity, prompts need to be sent when people are lacking physical activity. In addition, people may become passive and overly rely on prompts to motivate them. This is counterproductive because prompts are supposed to assist individuals in becoming more physically active rather than entirely dictating individuals’ physical activity. Periodic prompts can encourage people, but suffer dependency and flexibility concerns. Therefore, the development of automated reminders that are based on individuals’ situations may be helpful for better prompt applications.

The exercise performance situation involves several degrees of uncertainty and imprecision. Sufficient physical activity recommended by World Health Organization (WHO) is stated as “at least 150 min of moderate-intensity physical activity throughout the week, or at least 75 min of vigorous-intensity physical activity throughout the week, or an equivalent combination of moderate- and vigorous-intensity activity” [6]. Exercise performance, including amount of physical activity and days spent exercising, are basic elements for automated prompting decisions. A person may exercise in different ways to achieve sufficient physical activity such as doing a lot of physical activity at a time but exercising on only a few days. A person may also take several days of exercise to achieve sufficient physical activity. The description of achieving sufficient physical activity uses linguistic terms that are imprecise and vague. To make decisions involving imprecision and uncertainty, fuzzy logic is a way to model and make decisions using natural language. Fuzzy logic is an effective way to quantitatively represent and process subjectivity in real-world applications [7,8,9]. The if-then rules of fuzzy logic are widely used to represent knowledge and experience [7,8]. Prompt decisions can be inferred from fuzzy rules. Fuzzy logic is an easy computational method and a way of expressing uncertain information.

We propose a fuzzy logic-based prompting mechanism to help people manage their physical activity. Smartphones have communication functions, sensors, and user-friendly interfaces, which have the potential to be excellent prompting devices for improving physical activity. We used a smartphone as a sensing and prompting device to implement our fuzzy logic prompting system. The mechanism estimates exercise, including walking and running, in the pattern recognition stage. The accumulated activity effective index (AAEI) is proposed to analysis exercise performance based on pattern recognition in the activity effective analysis stage. The prompting decision can be made based on the activity effective analysis. The different stages of the prompting mechanism including pattern recognition and activity level estimation, activity effective analysis and accumulated activity effectiveness index, and fuzzy logic prompting mechanism is presented in the Materials and Methods section. The Results section demonstrates a simulation and real-world trial. Section 4 discusses the experiments and methods. Section 5 concludes the work.

## 2. Materials and Methods

**Figure 1:**
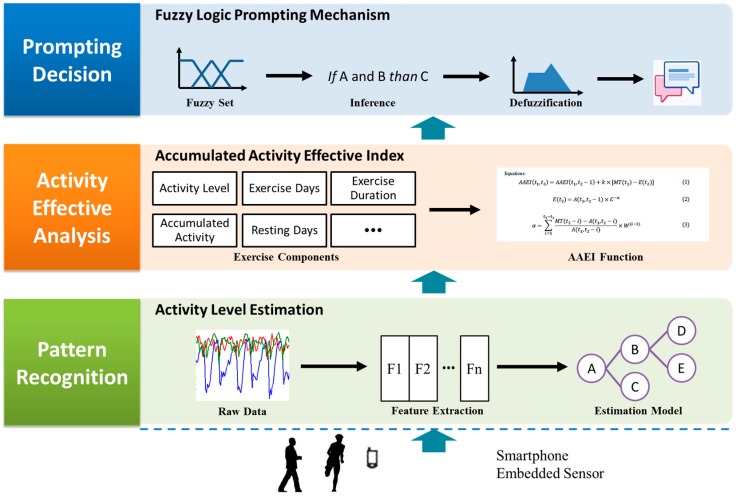
Platform of fuzzy logic prompting mechanism implemented in a smartphone.

The architecture of the proposed fuzzy logic prompting mechanism is illustrated in Figure 1. The prompting system uses fuzzy logic to decide whether prompts should be delivered based on an individual’s exercise performance. In our system, fuzzy logic is an approach to compute the degrees of sufficient physical activity. We also use experts’ knowledge to set rules for reasoning because there is no certain value to indicate the degrees of exercise performance [7,8]. We used a smartphone-embedded accelerometer, which was worn on a user’s left upper arm, to sense physical activity. The raw data sensed by the accelerometer were processed through an activity level estimation stage to estimate the level of activity performed by users. The activity level estimation comprises multiple components, which are the signal preprocessing, segmentation, feature extraction, and estimation model. Because walking and running are everyday physical activities, we estimated these two physical activities. Physical activity, such as people’s amount of physical activity and days spent exercising, are the basic elements for making decisions. However, a large amount of personal physical activity information may also complicate for automated systems and not be suitable for a mobile system. Consequently, we used an accumulated activity effective index to rate individuals’ physical activity based on a numeric index to enhance the decision-making of our prompt. The AAEI is a numeric indicator that estimates individuals’ exercise based on their activity levels and days of exercising [10]. The activity level was then used at the accumulated activity effective index stage to analyze exercise performance. The AAEI was proposed to estimate physical activity and generate a numerical index based on several components such as amount of physical activity and days spent exercising. Prompting decisions were made based on the AAEI results. The fuzzy logic prompting mechanism makes prompting decision once a day and delivers a prompt message that depends on exercise performance. Users can read the suggestion before exercising and receive a prompt if their physical activity is not sufficient.

### 2.1. Pattern Recognition and Activity Level Estimation

Physical activity can be measured as energy expenditure by using the metabolic equivalent of task (MET) as a unit to quantify activity level [11]. Activity level is increased with the intensity of physical activity. Sedentary intensity is defined as approximate 1 MET [11]. It is estimated that compared with sedentary intensity, a person’s caloric consumption is one to three times higher when being lightly active (1–3 METs), three to six times higher when being moderately active (3–6 METs), six to nine times higher when being vigorously active (6–9 METs) and more than nine times higher when being very vigorously active (>9 METs). Activity level can be determined using questionnaires, biomedical methods (ex. Doubly Labeled Water), and sensors [11,12]. Compared these methods, sensors have the advantages of being inexpensive, objective, and suitable for creating individual records for estimating activity level. The technique issues such as battery life and friendly user interface design are however challenges for sensor measurement.

**Figure 2:**
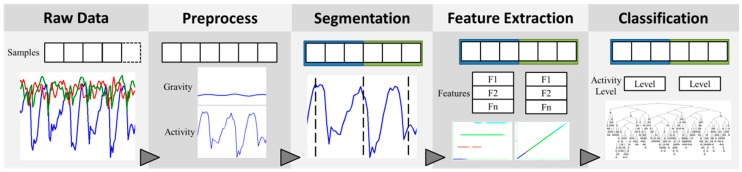
The multi-stage process of pattern recognition and activity level estimation.

The mechanism of pattern recognition and activity level estimation in the prompting system uses a multi-stage process comprising four processes, which are signal preprocessing, segmentation, feature extraction, and classification, as shown in Figure 2. The accelerometer embedded in the smartphone records the raw exercise data that contains gravity and motion acceleration. The sampling rate is 40 Hz. 

**Figure 3:**
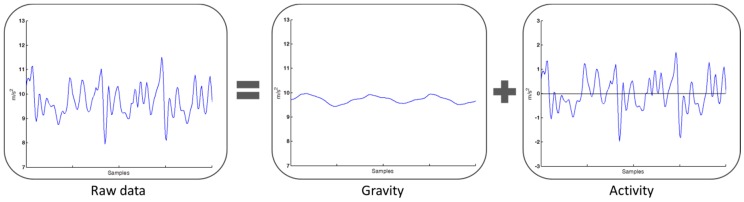
The raw data sensing by accelerometer contains motion acceleration and gravity.

Before estimating the activity level, the gravity should be separated. A low pass filter is used in the preprocessing stage to separate gravity and motion data, which are linearly combined in the raw data as shown in Figure 3 [13]. The low pass filter is a two-order elliptical infinite impulse response filter with cut-off frequency at 0.5 Hz. The filter can approximately to separate gravity and motion acceleration. The motion acceleration is then taken as the difference between the raw data and gravity. Then the body motion signal uses a window technique to divide the continuous motion signal into segments with a window size of two seconds. The window size impacts the activity level estimation. A smaller window size can detect physical activity faster and with reduced resources. A larger window size is suitable for detecting complex physical activity [14]. To develop a pattern recognition using smartphones for real-time application, the processing time and data buffer are constrained. In our system, two seconds provides a good trade-off between of estimation speed and accuracy [14], so we set a two seconds data buffer for segmentation.

The features are extracted from the motion acceleration segmentation to be a feature vector. Previous studies have generated many features for pattern recognition [11,13,15,16,17,18,19]. Time-domain and frequency-domain features are often generated to characterize information within time-varying signals [18]. Time-domain features are generated directly from segmentation of sensor data. Frequency-domain features are derived from frequency domain data which are transformed from sensor data. The Fast Fourier Transform (FFT) is widely used to transform the sensor data to frequency domain data [18]. To decrease the resources needed and processing complexity, we selected several time-domain and frequency-domain features. The features are generated and selected based on several well-known studies [10,11,13,15,16,17,18,19]. The feature vector contained the signal magnitude vector, signal magnitude area, maximum value of y- and z- axis, and the first three magnitude values and frequencies of the Fast Fourier Transform. The feature vector is used to classify the different activity level by Decision Tree algorithms [18]. The estimated activity level consisted of walking, fast walking, running, and being stationary, since walking, fast walking and running are ordinary physical activities and often performed by people. The activity categories and corresponding activity levels are defined in Table 1. 

**Figure 4:**
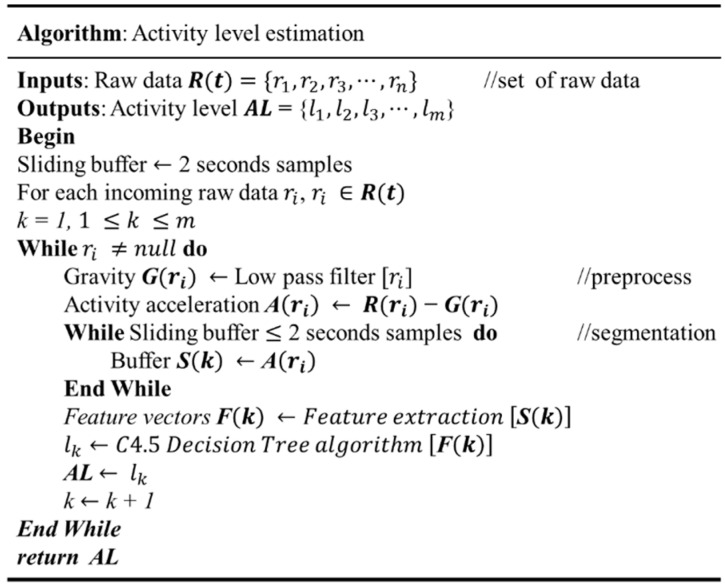
The activity level estimation procedure and algorithm.

The estimation model used C4.5 Decision Tree algorithm to identify various activity level categories. The C4.5 Decision Tree algorithm is one of the most extensively and successfully machine learning techniques used to classify categories in previous recognition problems [14,18]. Decision Tree algorithms have the advantages of ease of coding, fast prediction, and ease of model construction. To construct the activity level estimation model, we recruited seventeenth participants to perform walking, walking fast, and running on a treadmill. The participants walked and ran at different speeds at their own pace. The age of participants was 22.5 ± 2.3 years old. Participants wore the smartphone on their left upper arm to collect the motion data, which are used to construct the C4.5 Decision Tree algorithm. Participants were asked to perform walking, and walking fast at speeds from 1 to 7 km/h. Running was performed at speeds from 6 to 9 km/h. Each speed was maintained for at least 2 min in increments of 1 km/h. In the training phase, a Decision Tree model with a labeled feature vector is used to build the activity level estimation model. The activity level estimation model is validated by 10-fold cross validation. The precisions of the estimation models of light, moderate, vigorous, and very vigorous intensity are 94%, 81%, 88%, and 74%, respectively. The generated estimation model is then implemented in a smartphone to estimate activity levels. The activity level estimation algorithm is presented in Figure 4. 

The activity level estimation algorithm is coded in an Android smartphone. The raw data is time sequence data incoming in real-time. To reduce resource needs and processing time, the algorithm estimates the activity level for each segment. The activity level output is time sequence data based on the duration of the recording time. There is consecutive time sequence data for each estimation. The amount of physical activity is the activity level multiplied by the time duration. The return of time sequence activity level is the basic context for prompting a decision.

### 2.2. Activity Effective Analysis and Accumulated Activity Effective Index

**Figure 5:**
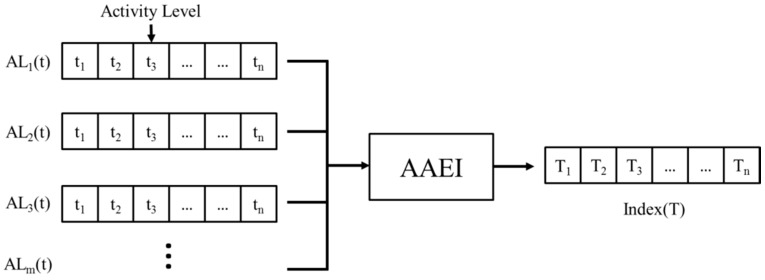
An accumulated activity effective index is estimated based on the time sequence activity level.

The AAEI is a stage to analyze exercise performance for mobile and real-time applications. The activity level is time data sequence which is estimated by the pattern recognition and activity level estimation stages. The activity level is given by the consecutive time sequence data when the system monitors the exercise performed by a user. The user may have exercised several times in one day. Each time may have different exercise durations. The days spent exercising and resting days may change according to the user’s personal situation. The amount of physical activity and accumulated amount of physical activity may vary while the user exercises in a period of time. Therefore, the consecutive time sequence activity level data are numerous. The system needs a lot of resources to record and analyze this data if we directly make prompting decisions based on these abundant time sequence data. Figure 5 shows how AAEI can combine several time sequence data into one time sequence dataset. AAEI can summarize activity level with an index. AAEI is used to analyze time series activity level data and reduce the complexity of the activity level.

The AAEI can indicate the extent to which users perform physical activity. The AAEI is designed as a numeral indicator that reveals the exercise performance of users after the pattern recognition stage. As shown in Figure 5, the consecutive time series activity level, $AL_{m}\left(t\right)$, can be summarized by AAEI to an set of indexes. The time unit of Index(t) is days, that is we measure exercise performance of users per day. The features of the AAEI are as follows: (a) the AAEI is related to the amount of physical activity accumulated in seven days. The AAEI increases if physical activity increases, remains constant if physical activity is constant, and decreases if physical activity decreases; (b) the AAEI is related to resting days. The AAEI decreases if the number of resting days increases; (c) the AAEI decreases more during periods of continuous rest than during intermittent rest; (d) the AAEI decreases less at rest if users had exercised before resting; and (e) the AAEI level is near zero if users rest for more than 7 days. The AAEI is used for both providing feedback to users and making prompt decisions. The AAEI and related parameters (Table 2) are described as follows [10]: Equation (1) shows how the AAEI index ($I\left(t_{1},t_{2}\right)$) is estimated from a previous AAEI index ($I\left(t_{1},t_{2}-1\right)$), the current amount of physical activity ($MT\left(t_{2}\right)$), and the threshold ($E\left(t_{2}\right)$). The threshold is used to decrease the AAEI index when there is a lack of physical activity. The threshold is determined by the accumulated amount of physical activity as shown in Equation (2). Equation (3) shows the estimation coefficient (α) of the accumulated amount of physical activity:

(1) $$I\left(t_{1},t_{2}\right)=I\left(t_{1},t_{2}-1\right)+k\times MT\left(t_{2}\right)-E\left(t_{2}\right)$$

(2) $$E\left(t_{2}\right)=A\left(t_{1},t_{2}-1\right)\times C^{-\alpha }$$

(3) $$\alpha =\overset{t_{2}-t_{1}}{\underset{i=1}{\sum }}\frac{MT\left(t_{2}-i\right)-A\left(t_{1},t_{2}-i\right)}{A\left(t_{1},t_{2}-i\right)}\times W^{\left(i-1\right)}$$

### 2.3. Fuzzy Logic Prompting Mechanism

**Figure 6:**
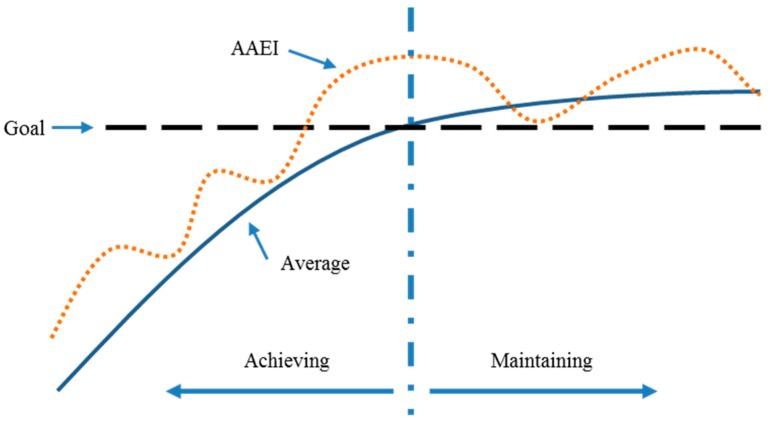
The principle of prompting decision making. The AAEI should be above average line in the goal achieving phase and the average line should above the goal in the maintenance phase.

The reminder makes prompt decisions based on AAEI values. Two decisions are as follows: (1) does the situation require a prompt; and (2) how much exercise should be prompted. The reminder’s decision-making principle is to help people achieve and maintain the goal of sufficient physical activity. The AAEI estimated for sufficient physical activity is 400–600 based on different exercise days and activity levels [10]. We choose 600 for the fitness goal because 600 is a high standard of sufficient physical activity. As shown in Figure 6, the exercise performance can be assessed and drawn as an AAEI dotted line. The average AAEI line represents the last 7 day average AAEI. The average AAEI line is estimated from individual users. To achieve this goal, the AAEI should be elevated above the average AAEI to increase the average AAEI. Prompts should be delivered if the AAEI is below the average AAEI during the achievement phase. After achieving the goal, the prompting system delivers prompts to help people maintain their fitness goals. The prompts are designed to be delivered if the average AAEI falls below the goal.

**Figure 7:**
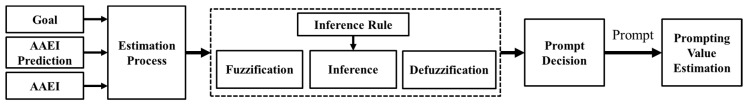
The framework of fuzzy logic prompting system.

The framework of the fuzzy logic prompting system is shown in Figure 7. When the system follows the prompting principles in the achievement and maintenance phases, the inference rule is applied. The inputs of the fuzzy logic prompting system are the goal, AAEI, and AAEI prediction. The AAEI is the estimated AAEI values recorded every day. The AAEI prediction is used to predict the physical activity status of a person. If the prompting system made a prompting decision based on the AAEI history, the prompts would be delivered after the situation occurred. For example, if the situation “user has failed to maintain the fitness goal” happens, then the prompts would then be delivered to the user. To deliver prompts before a potential failure situation happens, AAEI prediction is used to estimate possible failures. The AAEI prediction sets users’ physical activity at zero to estimate the tomorrow’s AAEI and takes into account the prompting decision. If the decision to prompt is positive, the prompting value will be estimated. The procedures of the fuzzy logic prompting mechanism are described as below.

#### 2.3.1. Convert Inputs into Fuzzy Sets

**Figure 8:**
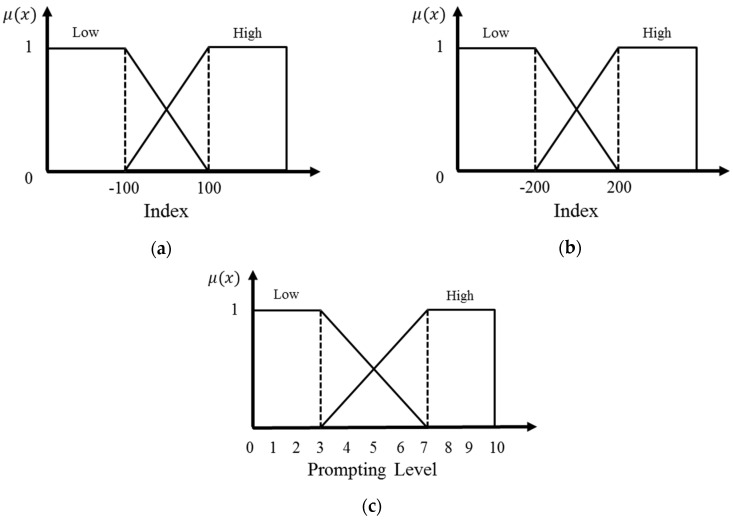
Fuzzy membership functions: (**a**) the fuzzy membership functions of *P1*, *P2*, and *P3*; (**b**) the fuzzy membership function of *P4*; (**c**) the fuzzy membership function of the prompting level.

Fuzzy membership function is usually determined by the perception of the linguistic terms, experimental data and simulation. Triangular and trapezoidal fuzzy membership functions have been widely used in the construction of fuzzy membership functions [7,8]. The trapezoidal fuzzy membership function is easy to construct and rational for two groups which are high and low as shown in the Figure 8. The prompting mechanism sets four estimated factors based on the inputs. The input factors and a prompting level (PL) are converted to matching to fuzzy membership functions. The fuzzy membership functions are shown as Figure 8. 

Based on Equations (1)–(3), the four estimated factors are described as follows:

(4) $$P1=\frac{1}{7}\;\left\{\left[\overset{5}{\underset{i=0}{\sum }}I\left(t_{1},t_{2}-i\right)\right]+I\left(t_{1},t_{2}\right)-E\left(t_{2}+1\right)\right\}-I\left(t_{1},t_{2}\right)+E\left(t_{2}+1\right)$$

(5) $$P2=G-I\left(t_{1},t_{2}\right)+E\left(t_{2}+1\right)$$

(6) $$P3=G-\frac{1}{7}\;\overset{6}{\underset{i=0}{\sum }}I\left(t_{1},t_{2}-i\right)$$

(7) $$P4=G-\frac{1}{3}\;\left\{\left[\overset{1}{\underset{i=0}{\sum }}I\left(t_{1},t_{2}-i\right)\right]+I\left(t_{1},t_{2}\right)-E\left(t_{2}+1\right)\right\}$$

where G=600, is the fitness goal. The principle of prompting rules is that the AAEI should be above the last seven days’ average AAEI in the achievment phase and the last seven days’ average AAEI should above the goal in the maintenance phase. The four estimated factors are estimated based on the principles. Factor P1 (predictive factor) and P2 (phase locating factor) are estimated based on the principle of “the AAEI should be above the seven day average AAEI” while factor P3 (phase factor) and P4 (maintenance factor) are estimated based on the principle of “the seven day average AAEI should be above the goal”. AAEI, AAEI prediction and goal are the elements of the estimation factors.

The factor P1 describes the difference between predictive seven days average AAEI and predictive AAEI. The “predictive AAEI” represents that the mechanism estimates the AAEI value of the user without any physical activity tomorrow. Based on Equation (1), the predictive AAEI becomes:

(8) $$I\left(t_{1},t_{2}+1\right)=I\left(t_{1},t_{2}\right)-E\left(t_{2}+1\right)$$

Predictive seven days average AAEI is the average of six known AAEI from today to five days ago and one predictive AAEI. Based on the Equations (1) and (8), the predictive seven days average AAEI, $PAI\left(t_{1},t_{2}\right)$, becomes:

(9) PAI(t1,t2)=17 [I(t1,t2−5)+I(t1,t2−4)+⋯+I(t1,t2)+I(t1,t2+1)]PAI(t1,t2)=17=17{[∑i=05I(t1,t2−i)]+I(t1,t2)−E(t2+1)}

Based on the Equations (8) and (9), the difference between predictive seven days average AAEI and predictive AAEI is shown as Equation (4). Following the principle of “the AAEI should above the seven day average AAEI,” the description “if user does not exercise tomorrow and the AAEI is below the average AAEI, the prompt should be delivered” can be constructed. Factor P1 estimates the difference between AAEI and the seven day average AAEI tomorrow, which is based on the principle. Factor P1 is converted to a fuzzy set as shown in Figure 8a.

Factor P2 estimates the difference between the goal and the predictive AAEI. It is a factor to estimate whether the user is achieving the goal or not. Considering both factors P1 and P2, the principle “the AAEI should be above the seven day average AAEI in the achievement phase,” can be measured. The degrees of factors P1 and P2 are the perception of the linguistic terms for inference.

Factor P3 estimates the difference between the goal and the seven day average AAEI. Factor P3 measures the level of the user in achieving the goal. Factor P4 estimates the difference between the goal and the predictive three days average AAEI. Considering both factors P3 and P4, the description “if the user’s average AAEI will below the goal, the prompt should be delivered” can be constructed. The description follows the principle of “the average AAEI should be above the goal in the maintenance phase.” The factor P3 is converted to match the fuzzy membership function as shown in Figure 8a. The factor P4 is converted to the match the fuzzy membership function as shown in Figure 8b. 

#### 2.3.2. Fuzzy Inference

Relations of P1, P2, P3, and P4 are constructed in a form of *if-then* rules. Rules are made based on the description in Figure 6. Two principles are made for inference: (1) AAEI should be above the seven day average in the achievement phase; (2) the seven days average AAEI should not be below the goal in the maintenance phase. The fuzzy inference then generates a mapping between input factors and output. Equation (10) describes the construction of the factors and prompting level in the form of *if-then* rules:

(10) $$R^{k}:\text{If}\;a\;\text{is}\;\mu_{a}^{k}\;\text{and}\;b\;is\;\mu_{b}^{k}\;\text{then PL is}\;\mu_{PL}^{k}$$

where a and b are estimated factors; $\mu_{a}^{k}$, $\mu_{b}^{k}$, and $\mu_{PL}^{k}$ denote the membership functions of a, b, and *PL*, respectively; $R^{k}$, k=1, 2, ⋯, n, is the kth rule in the inference rule.

For example, the rule 1 “If P1 is high and P2 is high then PL is high” is made based on the principle “AAEI should above the seven day average in the goal achievement phase”. The rule 5 “If P3 is high and P4 is high then PL is high” is made based on the principle “The seven day average AAEI should not below the goal during the maintenance phase”. The rules in the prompting mechanism are shown in Table 3.

The rules can be inferenced after the rules are made. The rule is inferenced by a fuzzy intersection (minimum) operation. The output of kth rule $\mu_{R^{k}}$ is described as follows:

(11) $$\mu_{R^{k}}=\mu_{a}^{k}\;\wedge \;\mu_{b}^{k}\;\wedge \;\;\mu_{PL}^{k}$$

To determine the inference result, the aggregations of rules use the fuzzy union (maximum) operation. The outputs of rules are aggregated with “and” in fuzzy logic. As described below, μR is the result of the fuzzy inference:

(12) $$\mu R={⋁}_{k=1}^{n}\mu_{R^{k}}$$

where *n* is the total number of rules. 

#### 2.3.3. Defuzzification and Prompting Value

The output of the fuzzy inference is a fuzzy set. Defuzzification is applied to convert the fuzzy result into a matching value. The membership function of the prompting level is shown in Figure 8c. The prompting system uses the Center of Area method for defuzzification. The Center of Area method calculates the center of gravity of the fuzzy inference. The prompting level can be calculated as follows:

(13) $$\text{PL}=\left(\overset{n}{\underset{i=1}{\sum }}w_{i}\mu_{\alpha }\left(w_{i}\right)\right)/\left(\overset{n}{\underset{i=1}{\sum }}\mu_{\alpha }\left(w_{i}\right)\right)$$

where n is the number of quantization levels of the output, $w_{i}$ is the amount of control output at the quantization level i, and $\mu_{\alpha }$ denotes to fuzzy membership function value in α.

The prompting level is obtained after defuzzification. The prompting level is the final result to measure the prompting need. We set a threshold at prompting level 5 based on the simulation and experimental data to determine whether a prompt should be sent. If the prompting message is decided to be delivered, the prompting value is then estimated as described in Equations (14)–(16). The prompting value (PV) is determined based on two elements: the value to achieve the seven day average AAEI and the value for progressing.

(14) $$\text{PV}=\frac{1}{7}\;\left[\overset{6}{\underset{i=0}{\sum }}I\left(t_{1},t_{2}-i\right)\right]-I\left(t_{1},t_{2}\right)\;+\;E\left(t_{2}\right)+progress\;value$$

(15) $$ps=\frac{1}{7}\;\left[\overset{6}{\underset{i=0}{\sum }}I\left(t_{1},t_{2}-i\right)\right]\;\times PL÷100$$

(16) $$\text{progress value}=\{\begin{array}{c} 50,\;ps\leq 50\\ ps,\;ps>50 \end{array}$$

The value to achieve the last seven day average AAEI is determined based on Equations (1–3). The value to achieve the seven day average AAEI is made based the prompting principles. The value to achieve the seven day average AAEI can ensure that the user stays physically active. The value for progressing is determined for increasing the physical activity. We expect that users can make progress based on their previous physical activity. We set the progress value to increase the physical activity based on the seven day average AAEI multiplied by the percentage of prompting level. The minimum progress goal is 50 because this value corresponds to moderately intense walking for 10 min.

### 2.4. Experiments

A set of experiment was conducted to validate the performance of the fuzzy logic prompting mechanism. A simulation model was used to test the decision-making by seeing if a person can always follow the prompts. Seventeen healthy participants capable of walking and running were recruited to conduct a 30-day real-world trial. The participants were asked to wear a smartphone on their left upper arm when they were walking and running. The participants in this experiment are different from the participants recruited in the training activity level estimation model. Before the end of the trial, we used an interview and questionnaire to analyze users’ experience. The results demonstrate how AAEIReminder performs in an ideal simulation model and a real-world trial.

## 3. Results

### 3.1. Simulation Model

**Figure 9:**
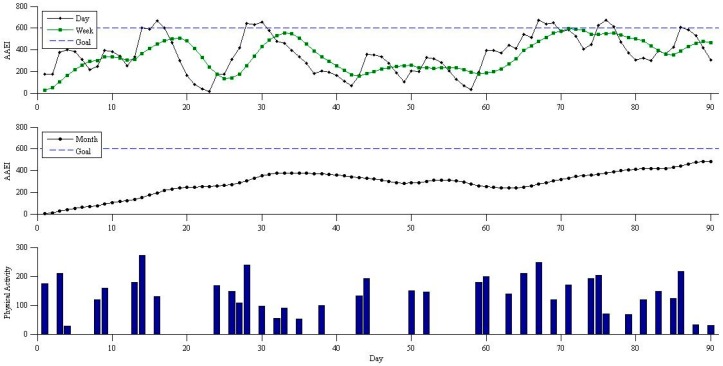
Physical activity and corresponding AAEI without prompting. Simulation model parameters- Intensity: 5 MET; Duration: 30 min; Frequency: 3.

**Figure 10:**
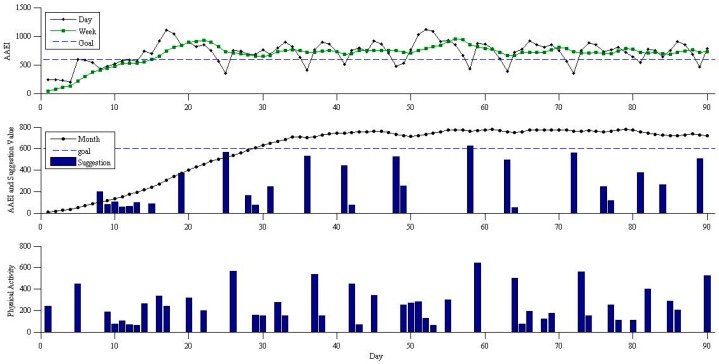
Physical activity and corresponding AAEI with prompting. Simulation model parameters- Intensity: 5 MET; Duration: 30 min; Frequency: 3.

A simulation model was used to validate the performance of the fuzzy logic decision-making. The simulation model can present an ideal performance of the fuzzy logic prompting mechanism. Figure 9 and Figure 10 show an example of the physical activity simulation results. Human activity can be described using a Poisson process based on previous studies [20]. We used a Poisson distribution to randomly generate physical activity. The simulation model shows insufficient physical activity with the simulation parameters: five MET as the average activity level and 30 min as the average exercise duration of each session with three sessions per week. The participant in this simulation was not sufficiently physically active to achieve the default goal. Figure 9 shows the AAEI, seven day average AAEI, 30 day average AAEI, and corresponding physical activity without prompting. The line of AAEI and the two average lines of AAEI show the trends corresponding to the simulated physical activity. The simulation model does not meet the goal which is set at 600. Figure 10 shows the simulation model with the fuzzy logic prompting mechanism. We assumed the suggestion values would be always followed if there is a prompt. The prompting value will replace the physical activity which is generated by the simulation model. The AAEI line and the two AAEI average lines in Figure 10 can stay in a region above the goal.

### 3.2. Performance of the Fuzzy Logic Prompting Mechanism

**Figure 11:**
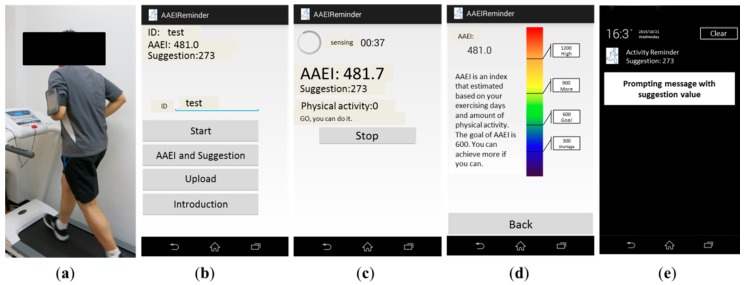
(**a**) The mobile phone is placed on the left upper arm while doing physical activity; (**b**) the screenshot of AAEI feedback and prompting suggestion before physical activity; (**c**) the screenshot of AAEI feedback and prompting suggestion when doing physical activity; (**d**) an introduction to the AAEI value for the user; (**e**) a delivered prompting message.

We recruited seventeen participants (seven females, ten males) to validate the performance of our fuzzy logic prompting mechanism. The group age was 27.2 ± 8.6 years old. In the real-world trial, the smartphone was worn on the left upper arm, as shown in Figure 11a, for convenience in reading the prompts. The AAEI can be used for goal-setting in the behavior motivation application. The user can receive a numeric index as feedback to evaluate his physical activity. Figure 11b shows the start page for receiving AAEI as feedback before engaging in physical activity. The participant could start to record the physical activity, as shown in Figure 11c, and read feedback provided in real-time to determine how much physical activity had been performed. The participant can read the introduction of the AAEI value as shown in Figure 11d. Figure 11e shows the prompting message if the prompt is delivered. The prompt is delivered once a day in the morning if determined by the system to be needed.

**Figure 12:**
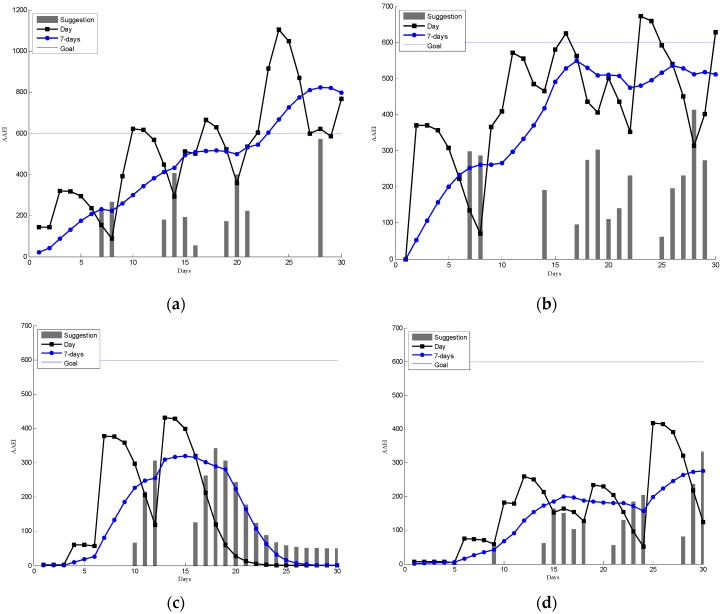

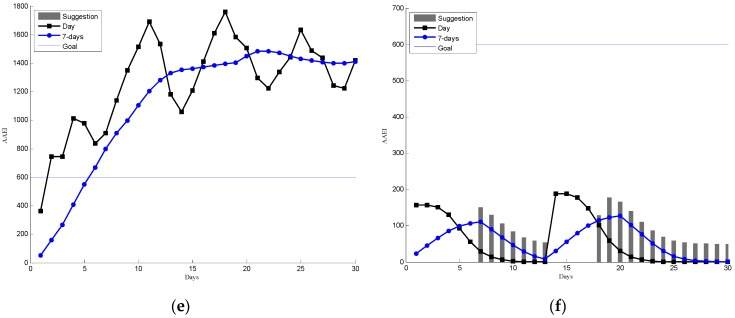
Different cases of recorded AAEI and prompting values in the real-world trail. (**a**) A case increase physical activity to achieve the goal; (**b**) a case increase physical activity near the goal; (**c**) and (**d**) two different cases that exercises sometimes; (**e**) a case achieve the goal without any prompts; (**f**) a case do little physical activity.

Figure 12 shows the participants recorded AAEI and prompting values in the real-world trial. The gray bar represents the prompting value. The prompting mechanism can automatically send prompting messages to the participants. The minimum prompting value is 50. The participants can increase or maintain sufficient physical activity if they follow the prompts. The participants can decide whether to follow the prompts in the trial. 

Figure 12 shows different cases in the experiment. Some cases increase their physical activity with the prompts, such as Figure 12a,b. Some cases exercise sometimes such as Figure 12c,d. Some cases can achieve the fitness goal without prompt such as Figure 12e. Other cases do little physical activity and often receive the prompts such as in Figure 12f. The participants can be divided into three groups based on the results of the experiment in the real-world trial. The participants can be classified into a group based on their last seven days average AAEI values. Table 4 shows the three groups with prompt delivering times and prompt following times. A prompt is defined as followed if a user exercises after receiving a prompt.

### 3.3. User Experience 

We recruited the participants to analyze the user experience of the fuzzy logic prompting mechanism after the trial. The questionnaire used 5 levels to score the user experience. Score 1 is for strongly disagree; Score 2 for disagree; Score 3 for normal; Score 4 for agree; Score 5 for strongly agree. The questions are listed in Table 5.

## 4. Discussion

This paper proposed a fuzzy logic prompting mechanism based on pattern recognition and AAEI using a smartphone-embedded sensor to automated deliver prompts. Smartphones have sensors, user-friendly interfaces, and processing units which is widely and easily used by people. Smartphones are suitable for the fuzzy logic prompting mechanism based on the pattern recognition and AAEI to validate the performance. However, pattern recognition using smartphone-embedded sensors is not an easy task. Gyroscopes can increase the activity monitoring accuracy [18,19]. However, the sensors embedded in smartphones sometimes do not have gyroscopes for activity monitoring. The sensor wearing position also affects the pattern recognition accuracy. The wearing position of smartphone is limited when the user is exercising, because a convenient and comfortable wearing position must take into consideration. Otherwise the user may not use the system even if the system is useful. The development of pattern recognition using smartphones to monitor other activities may be a challenge in future works.

The AAEI method in the activity effective analysis stage aggregates time sequence activity monitoring results to an index. The AAEI method is based on previous study [10] and it is possible to change the value of some parameters of the AAEI, however, the AAEI results may also be changed. These changes may affect the setting of the membership functions or inference rules in the fuzzy logic prompting mechanism. The default parameter values may not be the best for activity effective analysis. The value of parameters may also be changeable based on individual status. It is worth checking after the prompting mechanism is deployed in the real world and more data collected.

The simulation model with fuzzy logic prompting mechanism achieves an ideal situation in maintaining fitness goals if a participant can always follow the prompts. The simulation model without prompts in Figure 9 shows that the goal of 600 can be achieved only a few times due to insufficient physical activity. Compared with the results of simulation without prompting, the physical activity with prompting mechanism in the simulation is maintained at a sufficient level for an average of 30 days and an average of seven days. The prompts delivery time follows the prompting principle and is based on the simulated situation. The prompts can be delivered before the physical activity becomes insufficient. The prompting value is useful for achieving or maintaining a fitness goal. The simulation results show the prompting application delivered the prompting messages based on the exercise performance to achieve and maintain physical activity, and users can have sufficient physical activity if they can always follow the prompts.

However, humans cannot always follow the prompts for various reasons. Figure 12 shows the cases where users may sometimes follow the prompts to manage their physical activity. According to our observation, the participants can be divided into three groups based on their exercise performance. The first group usually performed high physical activity in the experiments. The prompts did not need to be delivered frequently because they can manage their physical activity. For example, Figure 12e shows the participant can achieve and maintain sufficient physical activity in the 30 days trial. Figure 12a–d shows the second group, that can sometime increase or maintain the physical activity with prompts. The participants in this group can sometimes follow the prompts to manage physical activity such as in Figure 12a,b. Sometimes the participants may not follow the prompts, such as in Figure 12d. The environment (e.g., cold or rain) and being busy with work are the main reasons that decrease the willingness to exercise. The participants in this group usually have sufficient physical activity (AAEI: 401–600) or near sufficient physical activity (AAEI: 250–400). The third group seems not take prompts into consideration when they manage their physical activity. Figure 12f shows an example where the participant has low AAEI despite many prompts.

Table 4 summarizes the three groups and the corresponding prompting times. The high activity group has the lower number of prompts compared to that of the medium activity group. This may indicate that the high activity group can manage their physical activity better than the medium group. The prompts followed times in the medium activity group are higher compared to that of the low activity group. It seems that prompts can help to increase physical activity. However, in the experiment this group is small. A bigger group and a long-term real-world trial are needed for a more detailed study.

The aim of the design of the prompting value is to achieve the average AAEI with a progress value based on the prompting principle in the fuzzy logic prompting mechanism. The prompting value may not be the best for a user. Because the physically condition and the environment were not taken into consideration, the user may not always be healthy enough to follow the prompts. Further study is needed to design the best prompting value. The proposed system is a context-based reminder that delivered prompts based on user’s physical activity. The system analyzes physical activity once a day based on the AAEI method. Therefore, the system makes a decision every day to decide whether the prompt should be delivered. The prompt is delivered once a day in the experiment. This prompt delivery once a day may not be the best frequency. The best prompt timing should consider interface design, user habits, the environment, and user context … etc. These could be topics for a future study for better system development.

The goal was set as 600 for sufficient physical activity. It may difficult for some participants to achieve the goal because 600 is a high standard for sufficient physical activity. The goal can set at 400 at least for sufficient physical activity according to the WHO recommendation. Thus some physically inactive participants may not find it difficult to achieve sufficient physical activity. The goal may also be changeable by users for their individual needs. Users may set the goal by themselves. In this paper, we set a stationary value to validate the fuzzy logic prompting mechanism.

The user experience was analyzed by the questionnaire. To the question of “AAEIReminder can help me to manage physical activity” the seventeen participants responded 3.6 on average. They thought that reminders can help them manage their physical activity. However, the participants thought that the display time of the prompting message was not good enough. This is not because the prompting mechanism did not show the prompting message before possible insufficient physical activity, but because the interface of the prompting message was not considered friendly enough. The prompting message is delivered once a day. We assume that the prompting message will be read when participants use for the first time their smartphones. Some participants thought the prompting message should be delivered more times if they need to take exercise or to be delivered at a specific time. This can be a future study topic for friendly interface design. The prompting value is designed based on participants’ previous exercise performance situation. The participants reported that the prompting value can be a reference for exercising. It is a useful for participants to be aware of their physical activity status. However, the participants also reported that they may not do more physical activity than the prompted value. The prompting value maybe cause participants to become passive. The cardiopulmonary function is also different between people. Increasing the prompting value is not a good way to help users manage their physical activity. Future study is needed to design a better prompting function.

## 5. Conclusions

We propose AAEIReminder, a fuzzy logic prompting mechanism based on pattern recognition and AAEI using smartphone-embedded sensor to deliver prompts. We implemented the mechanism in a smartphone for a prototype test. In the simulation, the prompts could be delivered automatically and help the users achieve or maintain sufficient physical activity levels if the users can always follow the prompts. In a real-world trial, the proposed mechanism was implemented and received positive responses from participants. The AAEIReminder, a fuzzy logic prompting mechanism based on the pattern recognition and AAEI is feasible for real-world use.

## Figures and Tables

**Table 1:** Activity Category and Corresponding Activity Level.

Activity		Activity Level
Walking		Light
Walking fast: 5–6 km/h		Moderate
	7 km/h	Vigorous
Running: 68 km/h		Vigorous
	≥9 km/h	Very vigorous

**Table 2:** Parameters of AAEI.

I: I≥0, an index	$\left(t_{1},t_{2}\right)$: time interval from $t_{1}$ to $t_{2}$, unit: days
k: k=1	$MT\left(t_{2}\right)$: $MT\left(t_{2}\right)\geq 0$, activity level (M) multiple duration (T) in day $t_{2}$
$E\left(t_{2}\right)$: $E\left(t_{2}\right)\geq 0$	$A\left(t_{1},t_{2}-1\right)$: $I\left(t_{1},t_{2}-1\right)$)/7
C: C=2	α: −2≤α<∞
i: i-th days before	W: W=0.5

**Table 3:** The rules of fuzzy inference.

Rule	Level of Estimated Factors		Prompting Level
	P1	P2	
Rule 1	High	High	High
Rule 2	High	Low	Low
Rule 3	Low	High	Low
Rule 4	Low	Low	Low
**Rule**	**Level of estimated factors**		**Prompting level**
	**P3**	**P4**	
Rule 5	High	High	High
Rule 6	High	Low	High
Rule 7	Low	High	High
Rule 8	Low	Low	Low

**Table 4:** Three groups of exercise performance.

Group	High Activity	Medium Activity	Low Activity
**AAEI range**	>600	250–599	<250
**Participants**	4	5	8
**Prompts**	6.3 ± 5.2	15.4 ± 2.1	17 ± 1.5
**Followed**	3.5 ± 2.6	3.6 ± 2.2	2.1 ± 1

**Table 5:** User experience analysis.

Questions	Average Score
1. AAEIReminder can help me to manage physical activity.	3.6
2. The prompting message was shown when I need physical activity.	3.2
3. I feel easier to manage my physical activity after I used the AAEIReminder system.	3.7
4. I will try to match the prompting value when I took exercise.	3.7
5. I feel the prompting value is reductant; I only need a message to remind me to take exercise.	2.5
6. I will always take exercise if I receive a prompting message.	3.1
7. I will take more exercise compared to prompting value.	2.9

## Abbreviations

The following abbreviations are used in this manuscript:

WHO	World Health Organization
AAEI	accumulated activity effective index
MET	metabolic equivalent of task
FFT	Fast Fourier transform
